# Predictors of futile recanalization after endovascular treatment of acute ischemic stroke

**DOI:** 10.1186/s12883-024-03719-8

**Published:** 2024-06-17

**Authors:** Li-Rong Wang, Bing-Hu Li, Qi Zhang, Xu-Dong Cheng, Li-Jun Jia, Sen Zhou, Shu Yang, Jian-Hong Wang, Neng-Wei Yu

**Affiliations:** 1 University of Electronic Science and Technology of China, Chengdu, 610054 China; 2 Department of Neurology, Sichuan Provincial People’s Hospital, Chengdu, 610072 China; 3 School of Clinical Medicine, Southwest Medical University, Luzhou, 646000 China

**Keywords:** Acute ischemic stroke, Endovascular treatment, Futile recanalization, Neutrophil-to-lymphocyte ratio

## Abstract

**Objective:**

Endovascular therapy (EVT) is the most successful treatment for patients with acute ischemic stroke (AIS) due to large vessel occlusion (LVO) in the anterior circulation. However, futile recanalization (FR) seriously affects the prognosis of these patients. The aim of this study was to investigate predictors of FR after EVT in patients with AIS.

**Method:**

Patients diagnosed with AIS due to anterior circulation LVO and receiving EVT between June 2020 and October 2022 were prospectively enrolled. FR after EVT was defined as a poor 90-day prognosis (modified Rankin Scale [mRS] score ≥ 3) despite achieving successful reperfusion (modified Thrombolysis in Cerebral Infarction [mTICI] classification of 2b-3). All included patients were categorized into control group (mRS score < 3) and FR group (mRS score ≥ 3). Demographic characteristics, comorbidities (hypertension, diabetes, atrial fibrillation, smoking, etc.), stroke-specific data (NIHSS score, ASPECT score and site of occlusion), procedure data (treatment type [direct thrombectomy vs. bridging thrombectomy], degree of vascular recanalization [mTICI], procedure duration time and onset-recanalization time), laboratory indicators (lymphocytes count, neutrophils count, monocytes count, C-reactive protein, neutrophil-to-lymphocyte ratio [NLR], monocyte-to-high-density lipoprotein ratio [MHR], lymphocyte-to-monocyte ratio [LMR], lymphocyte-to-C-reactive protein ratio [LCR], lymphocyte-to-high-density lipoprotein ratio[LHR], total cholesterol and triglycerides.) were compared between the two groups. Multivariate logistic regression analysis was performed to explore independent predictors of FR after EVT.

**Results:**

A total of 196 patients were included in this study, among which 57 patients were included in the control group and 139 patients were included in the FR group. Age, proportion of patients with hypertension and diabetes mellitus, median NIHSS score, CRP level, procedure duration time, neutrophil count and NLR were higher in the FR group than in the control group. Lymphocyte count, LMR, and LCR were lower in the FR group than in the control group. There were no significant differences in platelet count, monocytes count, total cholesterol, triglycerides, HDL, LDL, gender, smoking, atrial fibrillation, percentage of occluded sites, onset-recanalization time, ASPECT score and type of treatment between the two groups. Multivariate logistic regression analysis demonstrated that NLR was independently associated with FR after EVT (OR = 1.37, 95%CI = 1.005–1.86, *P* = 0.046).

**Conclusion:**

This study demonstrated that high NLR was associated with a risk of FR in patients with AIS due to anterior circulation LVO. These findings may help clinicians determine which patients with AIS are at higher risk of FR after EVT. Our study can provide a theoretical basis for interventions in the aforementioned population.

**Supplementary Information:**

The online version contains supplementary material available at 10.1186/s12883-024-03719-8.

## Introduction

In recent years, there has been a decrease in the incidence of stroke globally. In the United States, it has dropped from the first cause of death to the fifth. On the contrary, there is a increased trend of morbidity, death, and disability of stroke in China. Ischemic stroke accounts for more than 60% of all strokes and still ranks first among the causes of adult deaths in China, bringing a huge economic burden to society and families [1–4]. For patients with acute ischemic stroke (AIS) due to large vessel occlusion (LVO) in the anterior circulation, endovascular therapy (EVT) has been shown to be an successful therapy [5], which has faster recovery and shorter hospitalization. However, it has been found that despite control achieved by EVT ([mTICI ] classification of 2b-3), up to 48.7% of patients have a poor 90-day prognosis (defined as a modified Rankin Scale [mRS] score ≥ 3), which was defined as futile recanalization (FR) [6, 7]. Thus, post-EVT FR is an important factor influencing the prognosis of patients receiving EVT and it is urgent to find independent predictors of post-EVT FR.

Recent evidence demonstrated that inflammatory response involved in the pathophysiology of ischemic stroke. Randomized control trials (RCTs) also demonstrated that anti-inflammatory therapies reduce the risk of vascular events, including stroke, in patients with coronary artery disease (CAD) [8]. Regarding inflammatory response, neutrophils and lymphocytes are the most important inflammatory cells and they are also can be easily monitored clinically. They play a role of surveillance and defence in a healthy organism. It has been reported that the ratio of neutrophils to lymphocytes (NLR) is associated with the prognosis and recurrence of ischaemic stroke [17]. For example, Oh et al. showed that among AIS patients undergoing EVT, NLR and MHR were higher and LMR lower in patients with poor prognosis, suggesting that NLR, LMR, and MHR may be independent predictors of prognosis in EVT patients [9]. However, these indicators of inflammation have not been extensively studied in null recanalisation, therefore, in our study, we investigated the relationship with FR recanalisation by analysing the neutrophil and lymphocyte derived ratios in post-EVT patients.

## Methods

### Research subjects

The present study prospectively enrolled patients with AIS due to anterior circulation LVO undergoing EVT between June 2020 and October 2022. Patients met the following inclusion criteria were included: (1) meet the diagnostic criteria for AIS [2] ; (2) underwent EVT alone or combined with intravenous thrombolysis; (3) TICI of 2b-3 after EVT [10]; (4) without rheumatoid immune disorders, severe hepatic or renal disorders, hematological disorders, or malignant tumors; (5) without any systemic infections that occurred at the time of specimen collection or 2 weeks before stroke onset; (6) complete 90-day follow-up.

### Procedures

EVT was selected for patients meeting the following criteria: (1) confirmed AIS and Bleeding ruled out by CT; (2) LVO confirmed by CTA or digital subtraction angiography; (3) within 6 h of onset, or more than 6 h of onset and meeting the DAWN study inclusion criteria [11, 12]; (4) obtaining informed consent from family members. Exclusion criteria: (1) confirmed intracranial hemorrhage or intracranial tumor on admission; (2) inability to take care of oneself; (3) previous psychiatric disorders that would interfere with neurologic evaluation; (4) any other condition deemed inappropriate by the investigator for this study.

The operators have more than ten years of experience and are highly regarded by experts in the field. They have obtained the relevant technical qualifications and have performed thousands of surgeries, with the surgical error rate controlled at one in a thousand or less. Intervention modalities included manual aspiration, stent-retriever thrombectomy, or both were applied based on the condition of individual patient. Bridging EVT was allowed, which was defined as patients who underwent EVT after receiving a standard dose of intravenous alteplase.

### Data collection

Firstly, we will send the blood specimens collected within 24 h of the onset of the disease to the Laboratory Department for analysis within 2 h (All those indicators of blood routine that we analysed were perfected before EVT. The lipids involved were partly preoperative and partly postoperative, but they were all done 24 h after onset). The instruments used are: DXC-800 automatic biochemistry analyzer and blood cell analyzer, Data collection included demographic characteristics of the patients, such as gender and age; comorbidities, including hypertension, diabetes, atrial fibrillation, and smoking history; ASPECT score; NIHSS score; procedure data (treatment type [direct thrombectomy vs. bridging thrombectomy], degree of vascular recanalization [mTICI], site of occlusion; post-EVT timely vascular reperfusion status; procedure duration time and onset-recanalization time); and laboratory parameters (lipid levels, lymphocytes count, neutrophils count, monocytes count, C-reactive protein, neutrophil-to-lymphocyte ratio [NLR], monocyte-to-high-density lipoprotein ratio [MHR], lymphocyte-to-monocyte ratio [LMR], lymphocyte-to-C-reactive protein ratio [LCR], and lymphocyte-to-high-density lipoprotein ratio [LHR]).

### Outcomes

The efficacy outcome was assessed using the modified Rankin Scale (mRS) score at 90 days, ranging from 0 (asymptomatic) to 6 (death). Futile recanalization was defined as poor 90-day function (mRS score ≥ 3). All patients were divided into the control group (mRS score < 3) and FR group (mRS score ≥ 3). The safety outcome was any intracranial hemorrhage within 24 h and symptomatic intracranial hemorrhage (24 h-sICH). The 24 h-sICH was defined according to the European Cooperative Acute Stroke Study III criteria [13].

### Statistical analysis

Data analysis was performed using SPSS 24.0 statistical software. Continuous data were presented as median and analyzed by Mann-Whitney U test between the two groups. The χ2 test or Fisher exact test was used for the analyses of categorical variables (hypertension, hyperlipidemia, diabetes mellitus, atrial fibrillation, smoking, and site of occlusion, etc.). Multivariate logistic regression analysis was used to analyze predictors of FR after EVT. *P* < 0.05 was considered a statistically significant difference.

## Results

### Baseline characteristics

**Fig. 1:**
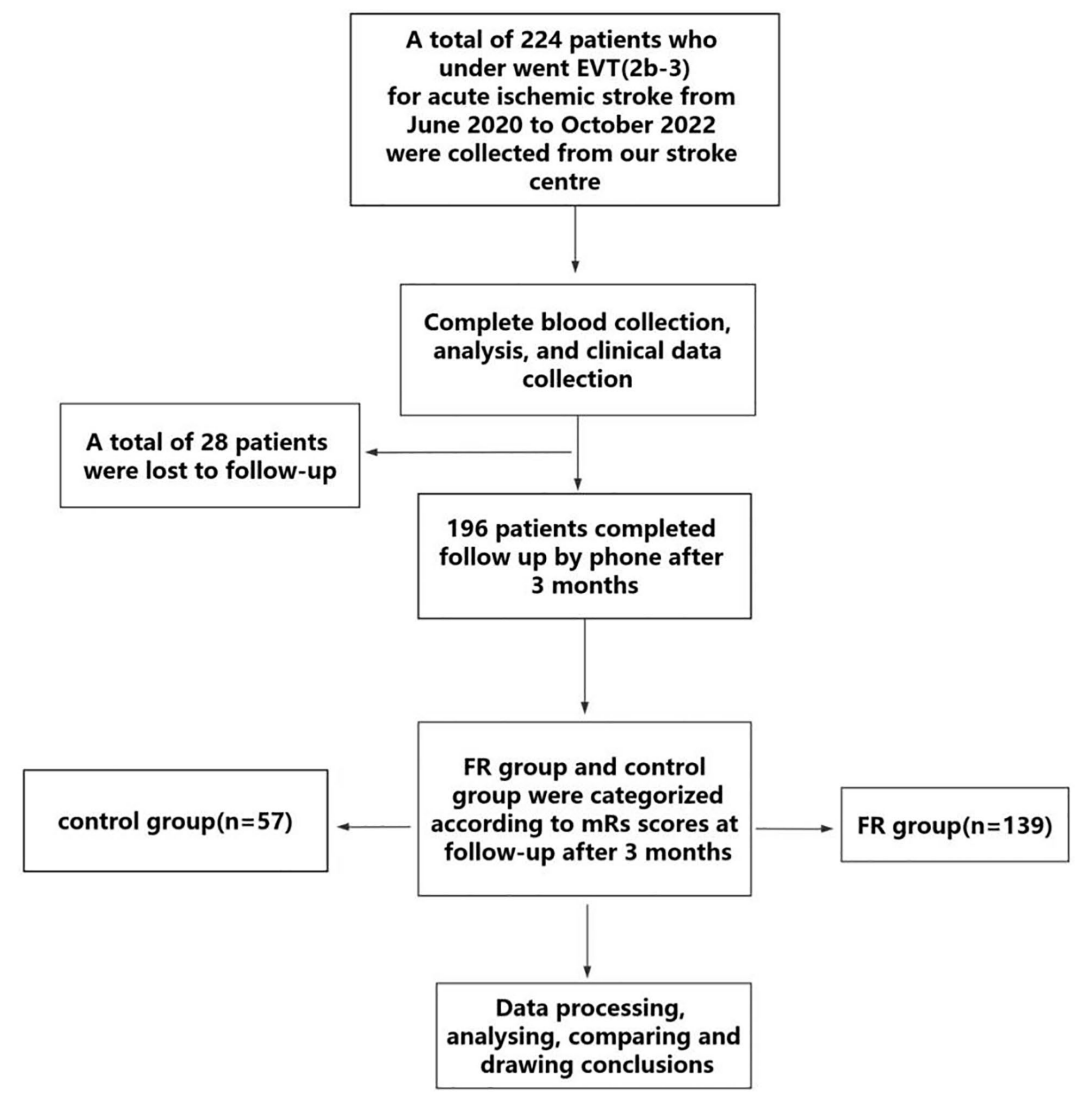
Study flow chart

A total of 224 patients who underwent EVT for AIS were collected, of which a total of 28 patients were lost to follow-up and 196 patients completed 90-day follow-up (Fig. 1). The median age (range) of the patients was 69.0 (28.0–95.0) years, with 106/196 (54.1%) being male. The median (range) baseline NIHSS score was 16 (3–35). The proportions of hypertension, diabetes, and atrial fibrillation were 48.5% (95/196), 19.9% (39/196), and 27.6% (54/196), respectively. Site of occlusion: intracranial internal carotid artery occlusion in 64/196 patients (32.7%); middle cerebral artery M1 segment occlusion in 120/196 (61.2%) patients, with approximately 12/196 (6.1%) of patients exhibiting tandem lesions. A total of 45/196 (23%) patients chose aspiration, 94/196 (48%) patients chose stenting and 57/196 (29%) patients chose aspiration combined with stenting. At 90 days follow-up, mRS 0–2 was observed in 29.1% (57/196) patients, and mRS 0–3 in 48.5% (95/196) patients. The 90-day all-cause mortality rate was 28.1% (55/196).

### Baseline comparison of control and FR groups

As shown in Table 1, a total of 139 patients were included in the FR group, and 57 patients were included in the control group. Age, percentage of patients with hypertension and diabetes mellitus, median NIHSS score, CRP, median neutrophil count, MHR and NLR were higher in the FR group than in the control group. The FR group had lower lymphocyte count, LMR, and LCR compared to the control group. There was no significant differences in platelet count, monocytes count, total cholesterol, triglycerides, LHR, high-density lipoprotein, low-density lipoprotein, sex ratio, smoking ratio, atrial fibrillation ratio, occlusion site ratio, onset-recanalization time and type of treatment between the two groups.

**Table 1:** Comparison of data between the FR and successful recanalization groups

	successful recanalization group (*n* = 57)		FR group (*n* = 139)	*P*-value
Age, years, median (range)		65.0 (29.0–90.0)	71.0 (28.0–95.0)	0.01*
Sex, male, n (%)		33 (57.9)	73 (52.5)	0.49
Hypertension, n (%)		19 (33.3)	76 (54.7)	0.007*
Diabetes, n (%)		6 (10.5)	33 (23.7)	0.035*
Atrial fibrillation, n (%)		14 (24.6)	40 (28.8)	0.55
Smoking, n (%)		18 (31.6)	31 (22.3)	0.17
ASPECT, median (range)		7.74(0–10)	6(0–9)	0.118
NIHSS, median (range)		12.0 (3.0–35.0)	18.0 (3.0–35.0)	0.00*
Site of occlusion				
ICA, n (%)		17 (29.8)	47 (33.8)	0.84
M1, n (%)		37 (64.9)	83 (59.7)	
Tandem, n (%)		3 (5.3)	9 (6.5)	
Direct thrombectomy, n (%)		19 (33.3)	37 (26.6)	0.35
Endovascular treatment modalities				
Stenting, n (%)		22 (38.6)	72 (51.8)	0.130
Aspiration, n (%)		18 (31.6)	27 (19.4)	
Aspiration + Stenting, n (%)		17 (29.8)	40 (28.8)	
CRP (mg/L) median (range)		3.24 (0.25–42.24)	6.14 (0.34-143.11)	0.00*
Lymphocytes (10^9/L) median (range)		1.34 (0.41–2.66)	0.96 (0.22–7.33)	0.00*
Neutrophils (10^9/L) median (range)		5.27 (2.67–14.43)	7.67 (0.42–27.89)	0.00*
Platelet count (10^9/L) median (range)		184.0 (55.0-367.0)	169.0 (49.0-415.0)	0.44
Monocytes (10^9/L) median (range)		0.39 (0.13–0.87)	0.44 (0.02–3.72)	0.06
HDLC (mmol/L) median (range)		1.27 (0.19-2.00)	1.25 (0.67–2.57)	0.39
Total cholesterol (mmol/L) median (range)		4.10 (2.04–6.34)	3.95 (1.16–7.40)	0.54
Triglycerides (mmol/L) median (range)		0.88 (0.35–3.09)	1.01 (0.39–5.92)	0.46
LDL (mmol/L) median (range)		2.35 (0.80–4.71)	2.07 (0.56–4.93)	0.19
NLR, median (range)		3.98 (1.06–18.78)	8.32 (0.16–71.16)	0.00*
MHR, median (range)		0.29 (0.0-0.74)	0.35 (0.0-2.53)	0.037*
LMR, median (range)		3.55 (1.20-11.41)	2.00 (0.16–24.50)	0.00*
LCR, median (range)		0.57 (0.03–4.47)	0.16 (0.00-14.50)	0.00*
LHR, median (range)		0.93 (0.0–2.0)	0.94 (0.0–5.0)	0.99
procedure duration time (minutes), median (range)		54.45 (13–115)	71.48 (16–345)	0.004*
onset-recanalization time (minutes), median (range)		425.03 (115–960)	427.63 (147–1032)	0.93

Note: ASPECT: Alberta Stroke Program Early CT Score. NIHSS: National Institutes of Health Stroke Scale; ICA: intracranial internal carotid artery; M1: M1 segment of the middle cerebral artery; CRP: C-reactive protein; NLR: neutrophil-to-lymphocyte ratio; MHR: monocyte-to-HDL ratio; LMR: lymphocyte-to-monocyte ratio; LCR: lymphocyte-to-C-reactive protein ratio; LHR: lymphocyte-to-HDL ratio; *: specific signs refer to significant *p*-value

### Multivariate regression analysis of predictors of FR

Factors that were different between the two groups were included in the multivariate logistic regression analyse: FR was the dependent variable; age, hypertension, diabetes mellitus, NIHSS score, CRP, lymphocyte count, neutrophil count, monocyte count, NLR, MHR, LMR, LCR were the independent variables. The results showed that baseline NIHSS score (OR = 1.06, 95% CI = 1.00-1.12, *P* = 0.048), NLR (OR = 1.37,95% CI = 1.005–1.86, *P* = 0.046), procedure duration time (OR = 1.020,95% CI = 1.008–1.034, *P* = 0.002) were independently associated with FR in Table 2.

**Table 2:** Results of multivariate regression analysis

Factors	β	OR	95%CI	*P*-vale
Age	0.210	1.022	0.990–1.060	0.250
Hypertension	0.650	1.920	0.700–5.270	0.210
Diabetes	0.188	1.190	0.260–5.490	0.820
procedure duration time	0.200	1.020	1.008–1.034	0.002*
NIHSS	0.065	1.060	1.000-1.120	0.048*
CRP	0.030	1.030	0.980–1.070	0.260
Lymphocytes	0.210	1.240	0.290–5.390	0.780
Neutrophils	-0.122	0.890	0.630–1.240	0.480
Monocyte	2.460	11.670	0.063–2148.400	0.360
NLR	0.310	1.370	1.005–1.860	0.046*
MHR	0.980	2.670	0.077–92.440	0.590
LMR	0.230	1.260	0.740–2.130	0.400
LCR	-0.230	0.800	0.540–1.170	0.240

Note: NLR: neutrophil-to-lymphocyte ratio; MHR: monocyte-to-HDL ratio; LMR: lymphocyte-to-monocyte ratio; LCR: lymphocyte-to-C-reactive protein ratio; CRP: C-reactive protein; NIHSS: National Institutes of Health Stroke Scale; *: specific signs refer to significant *p*-value

## Discussion

AIS imposes a huge economic burden on society due to its high morbidity and disability. The key to AIS treatment is blood flow reconstruction and stopping continued neuronal damage. With regard to revascularization, intravenous thrombolysis and EVT are the cornerstones of treatment of AIS [2]. Especially for patients with anterior circulation AIS due to LVO, EVT has been shown to be the most successful treatment [5, 14]. Clinically, FR occurred in up to 48.7% patients after EVT, being an important factor affecting the prognosis of patients after EVT [6]. Identifying risk factors for FR is important for preoperative patient screening and postoperative functional prediction. The present study found that NIHSS score and NLR were independently associated with FR after EVT.

FR is currently a hot topic of neurological research, but the mechanisms behind FR has not been fully clarified. Possible mechanisms include poor collateral circulation, distal embolization, early reocclusion, large hypoperfused areas, microvascular damage, impaired cerebral autoregulation and no-reflow phenomenon [15–17]. A recent meta-analysis with 2138 patients analyzing age, gender, baseline NIHSS, ASPECTS, hypertension, diabetes mellitus, atrial fibrillation, lipids, admission systolic blood pressure, diastolic blood pressure, glucose, site of occlusion, intravenous thrombolysis, TOAST typing, onset-to-recanalization time, perforation-to-reconvalescence time, and postprocedural symptomatic intracranial hemorrhage (sICH) showed that females, comorbidities, admission systolic blood pressure, glucose, occlusion site, non-bridging treatments, and postprocedural complications were predictive factors of FR [10]. Our study also examined age, comorbidities (hypertension, diabetes mellitus, atrial fibrillation, smoking), NIHSS scores, site of occlusion, surgery-related data (type of treatment, procedure duration time and onset-recanalization time) and laboratory markers (lymphocytes, neutrophils, monocytes, CRP, NLR, MHR, LMR, LCR, LHR), yielding that the NIHSS score, NLR, procedure duration time were higher in the FR group than in the control group. Further regression analyses also showed that high NIHSS score, NLR, procedure duration time were independently associated with the risk of developing FR after EVT.

Neutrophils play a key role in the innate immune system, reflecting the acute phase of the inflammatory response. The inflammatory response is usually not limited to the initial stages of infarction and can last from days to weeks. This continuing inflammation may lead to continued nerve damage and repair processes after infarction. Lymphocytes represent acquired immunity and induce autoimmune inflammation, especially playing a role in chronic inflammatory responses. In terms of individual components, both neutrophils and lymphocytes are involved in the entire process of stroke onset, progression, and prognosis: higher neutrophils are associated with stroke onset severity and ischemic stroke recurrence, whereas lower lymphocyte counts are associated with poor prognostic function [14, 18]. Neutrophil-to-lymphocyte ratio (NLR) integrates information of both innate and acquired immune responses, which has been seen as a biomarker of inflammation [19, 20]. It has been reported that NLR had more predictive value than neutrophils count alone, and can be considered an important indicator of systemic inflammatory status in Asian populations [21]. Previous study on ischemic stroke showed that NLR was significantly associated with severity of neurological deficit, poor primary functional prognosis, and ischemic stroke recurrence [22]. Our study showed that high NLR was associated with FR, which may reflect the possible role of neuroinflammation and abnormal immunomodulatory function in FR, and that targeting neuroinflammatory therapy may help to reduce the risk of FR after EVT.

Several limitations of this study should be acknowledged. Firstly, our data were collected from a single time point and a single center, further studies with data from different time periods and different hospitals are needed to validate our findings. Secondly, our study has not been able to confirm a causal relationship between NLR and FR, which needs to be confirmed by further validation. Thirdly, many postoperative complications such as aspiration pneumonia, may affect prognosis and were not collected and analyzed in our study, which may have had an impact on the results. Lastly, we do not routinely record and calculate volumes for each patient and do not perform 24 h and 7 days scoring. We will consider these data in later studies of the research. In conclusion, high NLR is associated with the risk of FR after EVT in patients with AIS due to LVO. NLR is expected to be an successful indicator for screening high-risk populations, and active attention and early intervention should be given to those at high risk of FR.

### Electronic supplementary material

Below is the link to the electronic supplementary material.

Supplementary Material 1

## Data Availability

Original data can be requested from the authors upon request.
