# Supplementary material for: Predictors of futile recanalization after endovascular treatment of acute ischemic stroke
Source: BMC Neurol. 2024 Jun 17;24:207. doi: 10.1186/s12883-024-03719-8 (PMC11181662; doi:10.1186/s12883-024-03719-8)
Supplement: Supplementary file 1 — Supplementary Material 1 [file 12883_2024_3719_MOESM1_ESM.doc]

Informed Consent Affirmation

Informed consent was obtained from all patients and their families for this experiment.
